# Effect of *Saccharomyces cerevisiae *var. Boulardii and β-galactomannan oligosaccharide on porcine intestinal epithelial and dendritic cells challenged in vitro with *Escherichia coli *F4 (K88)

**DOI:** 10.1186/1297-9716-43-4

**Published:** 2012-01-25

**Authors:** Roger Badia, Galliano Zanello, Claire Chevaleyre, Rosil Lizardo, François Meurens, Paz Martínez, Joaquim Brufau, Henri Salmon

**Affiliations:** 1 Institut de Recerca i Tecnologia Agroalimentàries, Animal Production, IRTA, Constantí, Spain; 2 Institut National de la Recherche Agronomique (INRA), UR1282, Infectiologie Animale et Santé Publique, F-37380 Nouzilly, Tours, France; 3 Immunologia Aplicada, Institut de Biotecnologia i de Biomedicina (IBB), University Autonomous of Barcelona, UAB, Bellaterra, Spain

## Abstract

Probiotic and prebiotics, often called "immune-enhancing" feed additives, are believed to deal with pathogens, preventing the need of an immune response and reducing tissue damage. In this study, we investigated if a recently developed β-galactomannan (βGM) had a similar protective role compared to *Saccharomyces cerevisiae *var. Boulardii (*Scb*), a proven probiotic, in the context of enterotoxigenic *Escherichia coli *(ETEC) infection. ETEC causes inflammation, diarrhea and intestinal damage in piglets, resulting in large economic loses worldwide. We observed that *Scb *and βGM products inhibited in vitro adhesion of ETEC on cell surface of porcine intestinal IPI-2I cells. Our data showed that *Scb *and βGM decreased the mRNA ETEC-induced gene expression of pro-inflammatory cytokines TNF-α, IL-6, GM-CSF and chemokines CCL2, CCL20 and CXCL8 on intestinal IPI-2I. Furthermore, we investigated the putative immunomodulatory role of *Scb *and βGM on porcine monocyte-derived dendritic cells (DCs) *per se *and under infection conditions. We observed a slight up-regulation of mRNA for TNF-α and CCR7 receptor after co-incubation of DC with *Scb *and βGM. However, no differences were found in DC activation upon ETEC infection and *Scb *or βGM co-culture. Therefore, our results indicate that, similar to probiotic *Scb*, prebiotic βGM may protect intestinal epithelial cells against intestinal pathogens. Finally, although these products may modulate DC activation, their effect under ETEC challenge conditions remains to be elucidated.

## Introduction

The infection by enterotoxigenic *Escherichia coli *(ETEC) is one on the most important causes of neonatal and post-weaning diarrhea (PWD) in piglets. ETEC causes significant morbidity and mortality, resulting in a large economic loses in the porcine industry. One of the most common ETEC in swine is serotype 0149 which carries the K88 (F4) adhesin that enables the attachment of the bacteria to the intestinal epithelium. ETEC colonizes ileum [1,2], penetrate the epithelium and its pathogenesis is ascribed to the production of different combination of heat-labile (LT) and heat-stable (ST) enterotoxins [3,4].

Antibiotic growth promoters (AGPs) have been long used in animal feeding to prevent neonatal and PWD in piglets. The use of AGPs increases the prevalence of bacteria resistant to antibiotics in farm animals and constitutes a potential risk of antibiotic resistance transference to human pathogenic bacteria, following to consumption of animal derived products [5]. The European ban of AGPs in animal production (EC 1831/2003) increased the need to develop new alternatives [6] to control and prevent animal colonization by pathogenic bacteria and somehow guarantee animal welfare and food safety.

Probiotics and prebiotics are interesting alternatives to AGP for animal feeding. They are believed to control pathogenic bacteria colonization and to enhance the mucosal immune system, resulting in a decreased pathogenic load and improving animal welfare [7]. The yeast *Saccharomyces cerevisiae *var. Boulardii (*Scb*) is a well-known probiotic with proven effects for the treatment and prevention of gastrointestinal diseases (see [8] for review). Typically, between 30-60% of *Saccharomyces *yeast wall is composed by polysaccharides [9] and specifically mannose and galactose mannans represent respectively more than 50 μg/mg of yeast dry mass [10]. Our center (Institut de Recerca i Tecnologia Agroalimentàries, IRTA) developed a highly rich β-galactomannan prebiotic (βGM) from the carob bean of the *Ceratonia silliqua *tree that as non-digestible food prebiotic ingredient may beneficially affect the host.

Intestinal epithelial cells (IECs) and dendritic cells (DC) of the gut are crucial for maintaining immunological tolerance to environmental, food antigens and commensal bacteria, but also to develop strong responses to invading pathogens when required [11,12]. Recent studies have demonstrated that IECs are far from being a simple physical barrier to the external environment. *Pathogen associated molecular patterns *(PAMPS) are recognized by *Pattern recognition receptors *(PRRs), such as *Toll-like receptors *(TLRs), expressed on IECs membranes [13], leading to the activation of proinflammatory pathways, as nuclear factor-κB (NF-κ-B) and activator protein 1 (AP1), related to cytokines and chemokines that coordinate the innate immune response [14].

Our work intended to establish an in vitro screening of an already known probiotic (*Scb*) and new developed prebiotic (βGM) to promote their use in animal feeding. First, we focused on antimicrobial activity and bacterial adhesion studies of these products on the IECs and how they may enhance an effective maintenance of the intestinal barrier. Furthermore, we studied their ability to modulate DCs which are pivotal for linking innate and adaptive immune response against pathogens. We were especially interested in the role of both cells types in cytokine and chemokine networks that regulate the homeostasis in the gastrointestinal tract [15].

## Materials and methods

### Intestinal epithelial cell culture

The porcine small intestine epithelial cell line IPI-2I (ECACC 93100622) was established from the ileum of an adult boar (SLAd/d haplotype) [16]. IPI-2I cells were maintained in DMEM (Invitrogen, Cergy Pontoise, France) supplemented with 10% FCS (Sigma-Aldrich, Saint-Quentin, France), 4 mM L-glutamine (Invitrogen), insulin 10 μg/mL (Sigma-Aldrich), 100 U/mL penicillin and 100 μg/mL streptomycin (Invitrogen). In all experiments, cells were cultured in 6-well plates (Falcon) to confluence. Before the addition of pre/probiotics and/or infection, cells were washed three times to remove antibiotics and culture media was replaced by DMEM media (Invitrogen) containing 4 mM L-glutamine (Invitrogen) and insulin 10 μg/mL (Sigma-Aldrich). Cells were used between passages 30-50 and periodically tested to avoid Mycoplasma contamination (MycoAlert^® ^Mycoplasma Detection Kit, Lonza).

### Probiotic and prebiotic preparation

Lyophilized *Saccharomyces cerevisiae *var. Boulardii (*Scb*, Biocodex, Laboratoires Montrouge, France) was rehydrated with 10 mL of DMEM and incubated for 30 min at 30°C. Then, yeast was counted with a Neubauer cell counter and methyl blue to exclude non-viable yeast. The yeast was added to the selected wells (multiplicity of infection, MOI = 3) and incubated overnight at 37°C and 10% CO_2_.

Commercially prebiotic Salmosan^® ^(patent WO2009/144070 A2, licensed by Industrial Técnica Pecuaria, ITPSA, Barcelona, Spain) contains more than 98% of β-galactomannan (βGM) which is a β-(1-4)-mannose backbone with branched galactose molecules (ratio galactose:mannose 1:4) [17]. Commercially prebiotic Salmosan^® ^also contain less than 1% of other polysaccharides such as xylose, fructose, arabinose, glucose, galactosamine and fucose derived from the carob bean gum and the seed of the *Ceratonia silliqua *tree. This is product is industrially treated to become soluble in gut. βGM is was diluted in DMEM (1 mg/mL), homogenized and incubated 30 min at 37°C. Immediately before the infection, βGM was added to each well at 10 μg/mL.

### Host cell-pathogen assay

Two pathogenic *E.coli *K88 (ETEC) strains were used in the host-pathogen assays. Initially, ETEC 56190 F4^+ ^(K88ad, O8:K87:H19, LT^+ ^and STb^+^) from INRA collection was used for cytokine and chemokine mRNA assays. For adhesion assays and cytokine protein secretion we used strain ETEC GN1034 F4^+ ^(K88^+^, LT^+^, STa^+ ^and STb^+^) provided by Dr Ignacio Badiola (Centre de Recerca en Sanitat Animal,CReSA, IRTA-UAB, Spain) which was more adherent to IPI-2I cells [18]. Both ETEC strains were preserved frozen in glycerol 15% at -80°C until their use. Before infection, 50 μL of ETEC were added to 20 mL of Luria-Bertrani media (LB) and cultured for 3-4 h at 37°C with 180 rpm rotational agitation (Multitron HT, Infors). For the infection, ETEC was used at exponential growth phase by determination of absorbance at 600 nm. ETEC was used at MOI = 10, previously determined by cytotoxic lactate dehydrogenase activity assay (Cytotoxicity Detection Kit Plus LDH, Roche). In vitro challenge lasted 3 h for gene expression and bacterial adherence studies or 24 h for supernatant cytokine determination. After host cell-pathogen co-culture, cells or supernatants were respectively sampled and stored until their analysis.

### Bacterial adherence assay

Protective effect of *Scb *and βGM was assessed by the adherence assay of ETEC on IPI-2I cells. Protocol was previously described by [19]. After the host-pathogen assay, supernatant was removed and cells were washed twice with sterile PBS to eliminate all non-adhered bacteria. Then, cells were homogenized with 1 mL of 0.1% Triton × 100 (Sigma-Aldrich) for 15 min. This solution was serially diluted in PBS and 100 μL (dilution 1 × 10^-3^) was plated in LB-Agar Petri dishes for 24 h at 37°C to enumerate the number of colony former units (CFU). The ability of ETEC GN1034 strain to adhere to IPI-2I cells was calculated as follows:

$$\text{Adherence (\%) = }\frac{\text{Adhered ETEC to IECs}}{\text{Total ETEC added/well}}\times 100$$

To determine adherence differences between experimental treatments, the relative percentage of adherence was calculated using next equation:

$$\text{Relative adherence (\%)}=\frac{\text{CFU/ml treatment}}{\text{CFU/ml control infection}}\times 100$$

### Isolation of mRNA and cDNA synthesis

Total RNA was isolated from homogenized cells using Trizol reagent (Invitrogen) and RNeasy MiniKit (Qiagen, Courtaboeuf, France). Then, RNA samples were treated with DNase I Amp Grade (Invitrogen) (1 U/μg of RNA). RNA concentration was determined by measuring optical density at 260 nm (OD260) and the RNA quality was assessed by calculating OD260/OD280 ratio and by capillary electrophoresis (Agilent 2100 Bioanalyzer, Agilent Technologies Inc., Santa-Clara, USA). A total of 1 μg of RNA was incubated in a final volume of 20 μL containing 2 μL dNTP (0.25 mM final each), 1.3 μL oligodT (133 pmoles/μL), 0.8 μL MuMLV reverse transcriptase (25 U/μL), 4 μL 5 × MuMLV buffer (Eurogentec, Liège, Belgium) and 1.9 μL of ultra-pure water (Gibco, Paisley, Scotland). The reaction was maintained for 90 min at 37°C and then heat-inactivated at 93°C for 5 min. The generated cDNA was stored at -80°C.

### Messenger RNA expression analysis using quantitative real-time PCR

Many mRNA and primer sequences have already been identified in pigs [20-22]. When genes were not described in this species, tBLASTn searches of the GenBank and PEDEblast ESTs databases, using known human and murine amino acid sequences, have been performed. These primers (purchased from Eurogentec) allowed the mRNA expression analysis of various genes involved in the innate immune response (Table 1). The qPCR was performed using cDNA synthesized as previously described [23]. Diluted cDNA (10X) was combined with primer/probe sets and IQ SYBR Green Supermix (Bio-Rad, Hercules, CA, USA) according to the manufacturer's recommendations. The qPCR conditions were 98°C for 30 s, followed by 37 cycles with denaturation at 95°C for 15 s and annealing/elongation for 30 s (annealing temperature, Table 1). Real time assays were run on a Bio-Rad Chromo4 (Bio-Rad). The specificity of the qPCR reactions was assessed by analyzing the melting curves of the products and size verification of the amplicons. Each qPCR reaction included a reverse transcription negative control (RNA sample without reverse transcriptase) to check the absence of genomic DNA. To minimize sample variation, we used identical number of cells and high quality RNA. Samples were normalized internally using simultaneously the average cycle threshold (C*q*) of Hypoxanthine PhosphoRibosyl-Transferase 1 (HPRT-1), Ribosomal Protein L 19 (RPL-19) and Tata Box Binding Protein 1 (TBP-1) [24] as references in each sample to avoid any artifact of variation in the target gene. These genes were selected as the reference genes because of their low variation between samples. A standard curve was generated using diluted cDNA. The correlation coefficients of the standard curves were > 0.995 and the concentrations of the test samples were calculated from the standard curves, according to the formula y = -M × *Cq *+ B, where M is the slope of the curve, *Cq *the point during the exponential phase of amplification in which the fluorescent signal is first recorded as being statistically significant above background and B the y-axis intercept. *Cq *values were used to calculate the qPCR efficiency from the given slope according to the equation: qPCR efficiency = (10[-1/M] - 1) × 100. All qPCRs displayed efficiency between 90% and 110%. Expression data are expressed as relative values after Genex macro-analysis with three reference genes (Bio-Rad, Hercules, USA) [25].

**Table 1:** Primer sequences and annealing temperatures of primer sets (°C), expected PCR fragment sizes (bp) and associated references.

Genes	Sense	Antisense	T° Annealing	Product length	Accession Number	Reference
**APRIL**	TGCTCACCCGTAAACAGAAG	TAAACTCCAGCATCCCAGAC	60	172	EST BP170456	[21]
**BAFF**	GAGAGCAGCTCCATTCAAAG	GCATGCCACTGTCTGCAATC	60	103	NM_001097498	[21]
**CCL2**	GTCACCAGCAGCAAGTGTC	CCAGGTGGCTTATGGAGTC	60	112	EF107669	[43]
**CCL17**	TGCTGCTCCTGGTTGCTCTC	ATGGCGTCCCTGGTACACTC	67	169	EST DB794536	[20]
**CCL20**	GCTCCTGGCTGCTTTGATGTC	CATTGGCGAGCTGCTGTGTG	66	146	NM 001024589	[21]
**CCR7**	AGGAGGCTCAAGACCATGAC	GATGCCGAAGATGAGTTTGC	62	147	AB090356	
**CXCL2**	TGCTGCTCCTGCTTCTAGTG	TGGCTATGACTTCCGTTTGG	60	171	NM_001001861	[21]
**GM-CSF**	GAAACCGTAGACGTCGTCTG	GTGCTGCTCATAGTGCTTGG	62	150	DQ108393	[21]
**HPRT1**	GGACTTGAATCATGTTTGTG	CAGATGTTTCCAAACTCAAC	60	91	DQ815175	[24]
**IL1α**	CCCGTCAGGTCAATACCTC	GCAACACGGGTTCGTCTTC	60	170	NM 214029	[43]
**IL6**	ATCAGGAGACCTGCTTGATG	TGGTGGCTTTGTCTGGATTC	62	177	NM_214399	[43]
**CXCL8**	TCCTGCTTTCTGCAGCTCTC	GGGTGGAAAGGTGTGGAATG	62	100	NM_213867	[21]
**IL10**	GGTTGCCAAGCCTTGTCAG	AGGCACTCTTCACCTCCTC	60	202	NM_214041	[22]
**RPL19**	AACTCCCGTCAGCAGATCC	AGTACCCTTCCGCTTACCG	60	147	AF435591	[21]
**TBP-1**	AACAGTTCAGTAGTTATGAGCCAGA	AGATGTTCTCAAACGCTTCG	60	153	DQ845178	[24]
**TGFβ**	GAAGCGCATCGAGGCCATTC	GGCTCCGGTTCGACACTTTC	64	162	NM 214015	[21]
**TLR4**	TGTGCGTGTGAACACCAGAC	AGGTGGCGTTCCTGAAACTC	62	136	NM_001113039	[21]
**TLR2**	ACGGACTGTGGTGCATGAAG	GGACACGAAAGCGTCATAGC	62	101	NM_213761	[21]
**TNFα**	CCAATGGCAGAGTGGGTATG	TGAAGAGGACCTGGGAGTAG	62	116	X54859	[21]

Reference genes are underlined

### Determination of cytokine production

Cytokine protein determination in the culture supernatant was performed by enzyme-linked immunosorbent assays (ELISAs). Host cell-pathogen assay was performed as described above but to avoid bacterial overgrowth Gentamycin 100 μg/mL (Sigma-Aldrich) was added to each well. Cell culture supernatant was collected after 24 h and stored at -80°C until analysis. Swine IL-6 and CXCL8 DuoSet ELISA from R&D Systems (Vitro SP, Spain) were used according to manufacturer's recommendations.

### Isolation of peripheral blood monocytes and differentiation of monocyte derived dendritic cells

Blood samples were obtained from 6 to 12-month old large white pigs at the slaughter house. Blood was collected into heparinised tubes and followed the protocol described by Pilon et al., [26] with few modifications. Briefly, peripheral blood monocytes (PBMC) were isolated by centrifugation (1000 *g *× 30 min) over Ficoll (d = 1.077, Histopaque, Sigma-Aldrich). Red blood lysing solution (Sigma-Aldrich) was used to remove remaining erythrocytes. Then, cells were resuspended in RPMI glutamax (Gibco, Invitrogen) containing 2.5% FBS, 1% Pen/strep and 50 μM of 2-β-mercaptoethanol (Sigma-Aldrich). Next, 150 × 10^6 ^cells/20 mL were plated in 150 cm^2 ^cellBind flasks (Corning, Afora, Spain) and incubated for 30 min at 37°C and 5% CO_2_. Then, flasks were washed with RPMI to remove all non-adherent cells (lymphocytes). To induce differentiation, monocytes were cultured with RPMI glutamax media containing 1% Pen/Strep antibiotic, 10% FBS, 50 μM β-mercaptoethanol and swine recombinant cytokines IL4 (100 ng/mL) and GM-CSF (20 ng/mL) (Biosource, Invitrogen) for 6 days at 37°C and 5% CO_2_. On day 3, fresh medium and cytokines were added at the same concentrations as previously.

### Dendritic cell phenotyping

After 6 days of culture, cells showed typical DC cell morphology, defined by large cytoplasmic cell mass and long dendrites. In addition, DCs were characterised as CD172a^+ ^(SWC3), SLA class II-DQ^+^, SLA class II-DR^+^, CD80/86^+^, CD14^mod ^and CD11R1^-^. Antibodies for cell surface markers CD172a/SWC3, SLA class II -DQ, SLA class II-DR and CD11R1 were provided by Dr J. Domínguez (INIA, Madrid, Spain). Antibody for CD14 determination was purchased from Acris Antibodies (AntibodyBCN, Barcelona, Spain) and for CD80/CD86 we used recombinant human Cytotoxic T-lymphocyte associated molecule-4/Fc fusion protein (CTLA4-Fc IgG1, Invitrogen). The FITC-conjugated anti-human immunoglobulin IgG1 or Zenon tricolor mouse IgG1 and IgG2a labelling kits (Invitrogen) were used for detection by flow cytometry (FACSCanto™ using FACSDiva™ software; BD Biosciences, San José, California, USA).

### Pathogen induced dendritic cell activation

After 6 days of culture, DCs were recovered and adjusted to 5 × 10^5^- 1 × 10^6 ^DC/well into 24-well plates. Then, DCs were incubated with *Scb *(MOI = 3) or βGM (10 μg/mL) and challenged with ETEC (MOI = 5). After 3 h of exposure, supernatants were discarded and cells were collected in Trizol reagent. Isolation of DCs mRNA and gene expression studies was performed as described above.

### Statistical analysis

All statistical analyses were performed using PROC GLM by SAS Software version 9.1.3 (SAS institute, Carey, NC, USA). Means for adhesion percentages, mRNA and protein secretion were considered in a factorial design 2 × 3 (2 infection level * 3 experimental treatments) with Duncann post-test grouping for analysis. The *P *value ≤ 0.05 was considered to be significant.

## Results

### Bacterial adhesion

**Figure 1:**
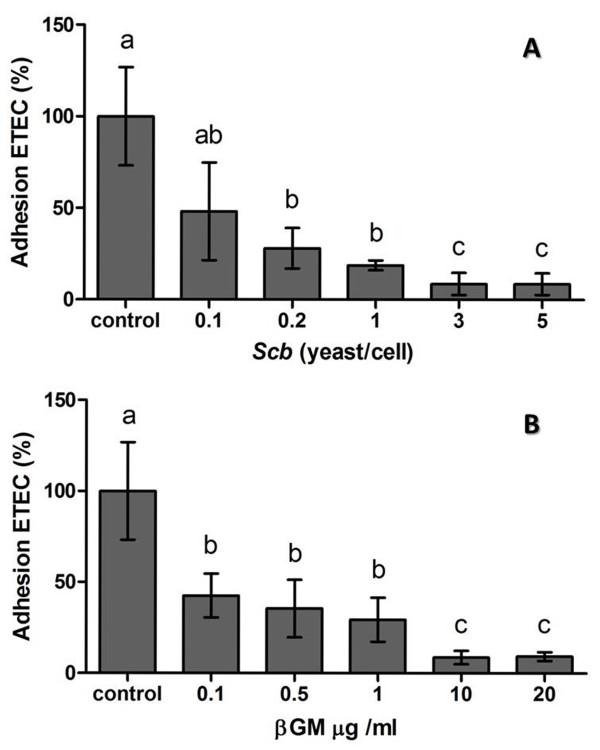
**Adherence of ETEC on pig intestinal IPI-2I cells in the presence of *Scb *or βGM**. Attachment of ETEC on IPI-2I cells co-cultured with *Scb *(A) or βGM (B) is inhibited in a dose-dependent manner. Data are presented as mean percentage ± S.D (*n *= 5) representative of 3 independent assays. Means with different superscripts (a, b, c) are significantly different (*p *< 0.05).

Adherence of ETEC on cell membrane of IPI-2I cells was measured to assess the ability of *Scb *and βGM to block ETEC fimbriae, inhibiting ETEC colonization of the intestinal tract. The adherence of ETEC K88 GN1034 (~5 × 10^6^-1 × 10^7 ^CFU) on pig intestinal IPI-2I cell line (~1 × 10^6 ^cells/well), was approximately 15% (data not shown). Presence of *Scb *(MOI = 3, Figure 1a) or βGM (10 μg/mL, Figure 1b) significantly inhibited ETEC attachment to 80% of control (*p *< 0.001) (Figure 1). These optimal doses of *Scb *or βGM were chosen for the following assays.

### Cytokine and chemokine mRNA expression of IPI-2I cells

**Figure 2:**
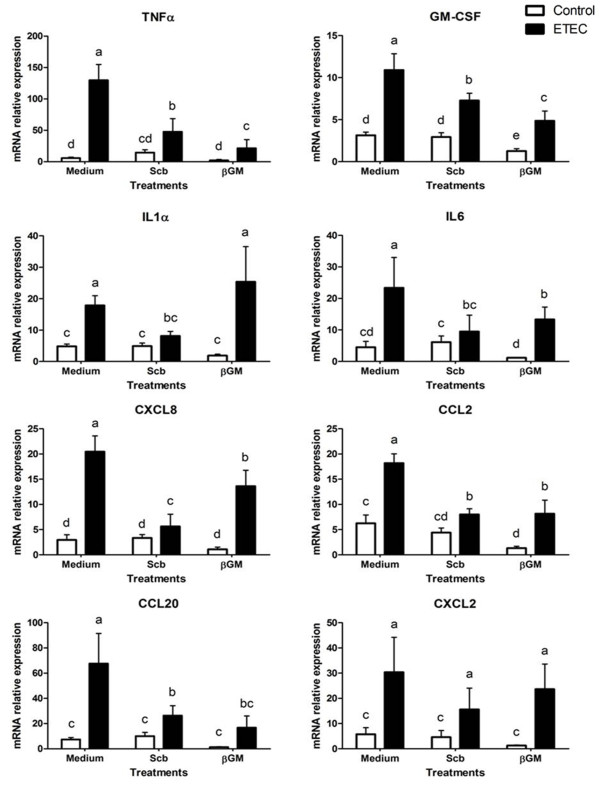
**Effects of *Scb *and βGM on cytokine and chemokine mRNA expression in IPI-2I cells cultured with ETEC**. IPI-2I cells (1 × 10^6^ cells/well) were co-cultured with *Scb *(3 yeast/cell) or βGM (10 μg/mL) with ETEC (1 × 10^7^ CFU/well) for 3 h. Data are presented as means of mRNA relative expressions ± SD (*n *= 6). Results are representative of 3 independent experiments. Means with different superscripts (a, b, c, d, e) are significantly different (*p *< 0.0.5).

To assess the preventive effect of *Scb *and βGM on the early immune response to ETEC, we studied the mRNA expression of several cytokines and chemokines. We also verified that *Scb *and βGM did not induce proinflammatory effect *per se *compared to control cells (Figure 2). As shown in Figure 2, IPI-2I cells cultured with ETEC showed a large up-regulation in the mRNA expression of genes related to inflammation cytokines and chemokines compared to the control group (*p *< 0.001). The bacterial challenge induced up to 20-fold increase in Tumor Necrosis Factor-α (TNFα) mRNA levels (*p *< 0.001) and 10-fold higher for chemokine (C-C motif) ligand 20 (CCL20, *p *< 0.001), whereas the increase was of lower magnitude, between 3- and 6- fold (*p *< 0.001, Figure 2) for Granulocyte/Macrophage Colony-Stimulating Factor (GM-CSF), CCL2, CXCL2, interleukins-1α, IL6 and chemokine (C-X-C motif) ligand -2 (CXCL2) and -8 (CXCL8).

The addition of *Scb *on IPI-2I cells induced a 50% inhibition of the ETEC-induced mRNA expression of TNFα, GM-CSF and CCL20 (*p *< 0.01) (Figure 2). Furthermore, *Scb *reduced the ETEC-induced mRNA expression for IL1α, IL6, CCL2 and CXCL8 genes to unchallenged control level (Figure 2). Similar to *Scb*, addition of βGM (10 μg/mL) down-regulated the ETEC-induced mRNA expression of TNFα, GM-CSF, CCL2, IL6 and CCL20 in infected cells (*p *< 0.01). Inhibition of CXCL8 was smaller in βGM treated cells (×1.5, *p *< 0.05, Figure 2) compared to *Scb *(×4). Finally, βGM did not inhibit IL1α ETEC-induced mRNA.

### Cytokine and chemokine secretion

**Figure 3:**
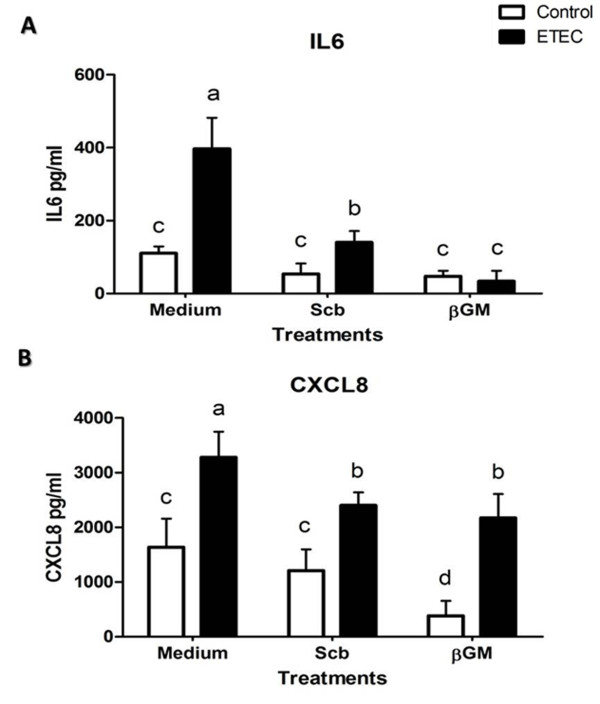
**Effect of *Scb *and βGM on IL6 and CXCL8 secretion induced by ETEC**. Cytokine IL6 (A) and chemokine CXCL8 (B) concentration in supernatants from IPI-2I cells (1 × 10^6^ cells/well) co-cultured for 24 h with ETEC (1 × 10^7^ CFU/well) is decreased by *Scb *(3 yeast/cell) or βGM (10 μg/mL). Data were presented as mean ± S.D (*n *= 6). Data are representative of 3 independent experiments. Means with different superscripts (a, b, c, d) are significantly different (*p *< 0.05).

To confirm the results for the mRNA expression, we studied the protein secretion of cytokine IL6 and chemokine CXCL8 by ELISA at 24 h after the infection. Similarly to the mRNA data, neither *Scb *nor βGM showed proinflammatory effects *per se *and ETEC infection caused a 3.8-fold up-regulation for IL6 concentration (*p *< 0.01) (Figure 3a) and a 1.8-fold increase for CXL8 compared to control wells (*p *< 0.01) (Figure 3b). Addition of *Scb *or βGM induced between 4- to 10- fold reduction for IL6 (Figure 3a) and a 1.4-fold decrease for CXCL8 (Figure 3b) (*p *< 0.05) without any statistical difference between both products.

### Modulation of mRNA expression of monocyte derived dendritic cells

**Figure 4:**
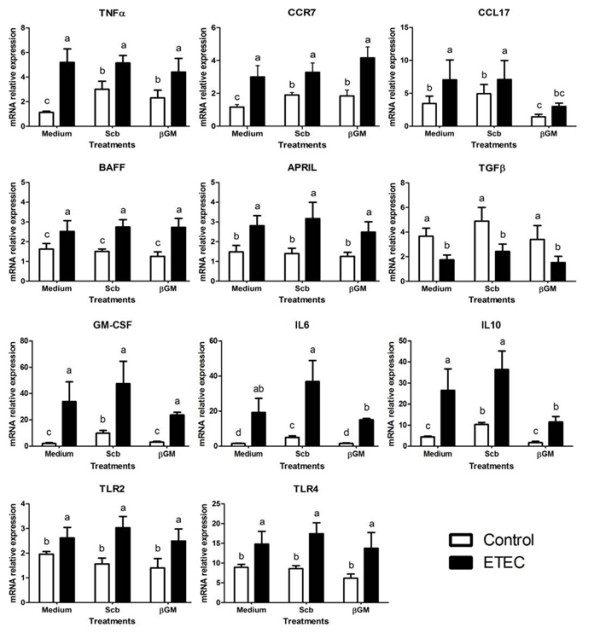
**ETEC-induced gene expression in porcine DCs co-cultured with *Scb *or βGM**. Relative mRNA expression of proinflammatory cytokines (TNFα, GM-CSF, IL6, IL10), chemokines (CXCL8, CCL17), receptors (CCR7, TLR2, TLR4) and regulatory factors (APRIL, BAFF, TGFβ) in DCs is enhanced by ETEC. Data were presented as mean ± S.D (*n *= 6). Treatments with different letters (a, b, c, d) mean *p *< 0.05.

Effects of *Scb *and βGM on DCs activation or maturation were determined by the exposure of these products to porcine monocyte-derived DCs with and without ETEC co-culture. The highest pathogen induced DC activation by ETEC was obtained at MOI = 5, previously determined by proinflammatory gene expression (mRNA) after 3 h of co-culture (data not shown). Our results showed that ETEC induced a 2.6 fold-increase of mRNA level of CCR7 and a 4.6 fold up-regulation for TNFα compared to control DC (Figure 4, *p *< 0.01). Gene expression for TLR4, TLR2, B-cell activating factor (BAFF), a proliferation-inducing ligand (APRIL) and CCL17 were between 1.5 and 2 fold-higher in ETEC-induced DCs than in unchallenged DCs (Figure 4, *p *< 0.01). The mRNA level for IL6 and GM-CSF were highly up-regulated (×13 and ×26 respectively) after ETEC co-culture, as well as for IL10 (×6) (Figure 4, *p *< 0.001). Therefore, we defined activated DC as high mRNA expression of CCR7 and TLR4 receptors, T-cell-independent IgA class-switch recombination (CSR) modulatory factors BAFF and APRIL; cytokines TNFα, IL6, GM-CSF, CCL17 and IL10.

The mere addition of *Scb *(MOI = 3) or βGM (10 μg/mL) triggered a slight but statistically significant up-regulation of TNFα (×2.7 and ×2 respectively) and CCR7 mRNA (×1.6) compared to the control with medium (Figure 4, *p *< 0.05). Both products also enhanced gene expression for GM-CSF (×5 and ×1.6). However, only *Scb *induced gene expression of IL6 (×3.3) and IL10 (×2.3) (Figure 4, *p *< 0.05).

The ETEC-induced mRNA expression of DCs co-cultured with *Scb *(MOI = 3) or βGM (10 μg/mL) was similar to infected control (Figure 4) for TNFα, CCR7, TLR4, TLR2, BAFF, APRIL and TGFβ genes. Therefore no synergistic or antagonistic effect was observed for these genes related to DCs activation. However, ETEC-induced mRNA expression of IL10 and CCL17 was 2.3-fold inhibited in βGM treated DCs (*p <*0.05, Figure 4).

## Discussion

After European ban of antibiotic growth promoters for animal feeding (EC 1831/2003), probiotics and prebiotics have been postulated as promising alternatives to AGPs in animal feeding [7]. This work studied the effect of a new commercial prebiotic compared to an already proven probiotic on porcine IECs and DCs in the context of an ETEC in vitro infection. Besides to provide selective advantages to microbiota and to exclude pathogens, prebiotics are believed to mimic the host cell receptor which the pathogen adheres. Mannose derivates are described to bind to Type I *fimbriae *or *pili *which contains multiple-subunits of lectins that bind to mannan units of the glycoproteins on the surface of host cells [27,28]. In that sense, Shoaf et al. [3], tested several commercially available non-digestible oligosaccharides (NDOs) with different molecular structures. The latter highlighted that galactoligosaccharides had higher inhibition of *E.coli *adherence on Caco-2 and Hep-2 cells in a dose dependent manner. Similar to NDOs, yeast probiotic properties, such as *Saccharomyces cerevisiae*, are specie- or strain- specific [29]. The ability of *Scb *to aggregate enterohaemorrhagic *E.coli *(EHEC) on their cell wall though type I fimbriae recognition was described by Gedek B. R [30]. However, ETEC K88 bears F4 fimbriae [31], which is described as mannose-resistant in the agglutination test with 0.5% D-mannose [32]. Nevertheless, all three variants of K88 adhesin expressed on the tip of the F4 fimbriae recognize carbohydrate structures expressed on host cell glycoconjugates [33] such as sialoglycoproteins, intestinal mucin-type glycoproteins and neutral glycosphingolipids [34]. Indeed, β-linked galactose residues have been found to be essential component in the attachment of K88 fimbriae to mucus K88 receptors [35,36]. Our results show that addition of *Scb *or βGM, reduces the adhesion of ETEC on the surface of porcine intestinal epithelial IPI-2I cell approximately to 80%. Together, these data confirm that products highly rich in galactose residues, like our βGM and *Scb*, may bind to K88 adhesin [27], suggesting their role as a prophylactic agent to gastrointestinal infections in pigs [37,38].

The enterotoxigenic *E.coli *K88 (ETEC) in vitro infection causes a proinflammatory transcriptional profile on porcine intestinal cells IPI-2I, upregulating gene expression cytokines TNFα, GM-CSF involved in proliferation and activation of neutrophils, IL1α, IL6 (acute phase reactions, proliferation and differentiation of macrophages and B-cells) as well as proinflammatory chemokines CXCL8 (neutrophil recruitment), CCL2 (MCP-1, Monocyte chemotactic protein-1), CXCL2 and CCL20 chemotactic for immature DCs involved in the bacterial uptake across the epithelial barrier. These results are in accordance to results of Eckmann et al., [39], but contrary to data published by Pavlova et al., [4] who found no differences in mRNA expression of cytokine TNFα and chemokine CXCL8 after in vitro attachment of different serotypes of ETEC on IPI-2I cells. However, response of IPI-2I cells under challenge conditions and mRNA expression of genes TNFα and CXCL8 has been already demonstrated in the gene expression study performed by Mariani et al., [40], as well as in different host-pathogen interaction studies where IPI-2I were infected by another Gram negative pathogen as *Salmonella *[41]; *Salmonella *lipopolysaccharide (LPS) [42] or even *Enteamoeba hystolytica *parasite [43]. The explanation for these contradictory results may be due the difference in cell culture conditions (see Mariani et al., [40]) or to the specificity of the ETEC serotype used in each study. In our model, presence of ETEC or their bacterial constituents such as LPS or flagellin induces a two to tenfold increase in mRNA expression of proinflammatory genes in IPI-2I cells compared to the non-infected cells. At the protein level, data for IL6 and CXCL8 in infected IPI-2I cells (Figure 3) are comparable to protein secretion of pig intestinal IPEC-J2 cells shown by Devriendt et al. [31], as a response of TLR5 signalling cascade upon recognition of ETEC F4 flagellin. Addition of *Scb *or βGM down-regulate ETEC-induced gene expression of TNFα, GM-CSF, IL6, CCL2, CXCL2 and CCL20, reducing the overall proinflammatory state caused by ETEC. This effect is not induced for mRNA of IL1α nor CXCL8 in βGM treated cells compared to the *Scb *treatment, but no difference was shown between IL6 and CXCL8 in the reduction of mRNA or protein expression.

In accordance to current data, probiotic *Scb *has been shown to secrete anti-inflammatory factors smaller than 10 kDa [44] that have inhibitory effects on mRNA expression of cytokine IL1-α, IL6 as well as chemokines CXCL2 and CXCL8 on IPI-2I cells co-cultured with ETEC [18]. These anti-inflammatory properties have been related to direct blocking of nuclear factor-kappa B (NFkB) and mitogen associated protein kinase (MAPK) activation [45] or by indirect neutralization of ETEC toxins [8]. Recently, Zanello et al. [46] demonstrated that *Saccharomyces cerevisiae *inhibition on proinflammatory transcripts in porcine intestinal cells IPEC-1 co-cultured with ETEC was associated to the decrease of ERK1/2 and p38 MAPK phosphorylation. Related to NDOs, beneficial role of βGM from partially hydrolyzed guar gum was described to prevent mucosal damage in dextran sulphate sodium (DSS) induced colitis in mice [47] by a decrease of TNFα mRNA/protein and neutrophil infiltration. Future studies should determine the role of NDOs and especially βGM on the molecular signaling pathways involved in regulation of IECs upon ETEC infection.

Probiotics and prebiotics are believed to be recognized by PRRs expressed on dendritic cells (DCs) located in the subepithelial dome (SED) and/or to extend their dendrites between epithelial cells to the lumen. We aimed to establish if optimal concentrations of *Scb *or βGM for IECs also modulate DC activation or maturation *per se *and/or under ETEC infection. In our work, brief incubation (3 h) with *Scb *or βGM induced a slightly activation of DC observed by an up-regulation of mRNA for TNFα and CCR7 coreceptor (Figure 4). Recently, Santander et al. [48] described that long exposure (~48 h) to βGM from *Caesalpinia spinosa *plant extracts induce phenotypic maturation of human DC characterized by CD83, CD86, CD206 and HLA-DR and increase mRNA for IL1β, IL6, CXCL8 and IL12p70 cytokines.

The ETEC-induced maturation of porcine DCs is mainly induced by flagellin, through Fcγ receptor ligation [49], among other bacterial constituents such as F4 and LPS [49,50]. We observed that ETEC up-regulated mRNA level of CCR7, receptor for CCL19 and CCL21, which provides DC signal for entry into the inductive sites of the adaptive immune system such as the lymph node. Furthermore, there was an increase in BAFF and APRIL, members of the tumor necrosis factor superfamily involved in T-cell-independent IgA class-switch recombination (CSR). Additionally, ETEC recognition by TLR4 induced up-regulation of this receptor and activated the gene expression of cytokines TNFα, GM-CSF, CCL17 and IL6. Finally, IL10 mRNA was increased in challenged DCs, probably to limit over-whelming specific and unspecific immune response and to avoid tissue damage [51]. Although no phenotypic study was performed in this work, published data [50] indicate that porcine monocyte-derived DC are expected to increase cell surface co-stimulatory molecules CD80/C86 and protein TNFα, IL6 and CXCL8 secretion after TLR ligand recognition.

In our study, co-incubation of porcine DCs with *Scb *or βGM and ETEC did not trigger significant differences on DCs maturation compared to infected control for the most of the genes studied. In contrast, Sonck et al. [52] found that effects of β-glucans on DC maturation differ according to their origin, while *Saccharomyces cerevisiae *derived β-glucan Macrogard induced mature phenotype determined by upregulation of DC activation markers (CD80/86, CD40 and MHC class II), curdlan enhanced expression of cytokines IL-1β, IL-6, IL-10 and IL-12/IL-23p40. Related to prebiotics, Wismar et al. [53] found that galactomannans from guar gum at high concentrations (~200 μg/mL) directly stimulate mice DCs and modulate DC maturation induced by microbial signals, such as LPS, leading to a synergistically increased TNFα and IL10 proteins and suppression of IL-12p70. Indeed, Sheng et al. [54] showed that mannan and mannan associated structures activate TLR4 signalling pathways in a dose dependent manner, induce DCs mature phenotype (CD40, CD80 and CD86) and up-regulate proinflammatory mRNA for IL1-β and TNFα cytokines as well as other Th1/Th2 cytokines. Considering that origin, structure and size of mannans or β-glucans is crucial to determine their immune regulatory role [53], differences in product concentration and time exposure may explain our contrary results.

The beneficial role of these products remains to be elucidated on DCs activation under ETEC infection, as well as for other cell types involved in the mucosal immune response, such as monocytes/macrophages or neutrophils. In addition to evaluate *Scb *and βGM in in vivo trials, research approaches as three-dimensional co-culture [55] or gut-loop intestinal models [21] may determine effect of *Scb *and βGM on the cross-talk of IECs, DCs and other cell types under ETEC infection.

To summarize, present findings describe the protective role of probiotic *Saccharomyces cerevisiae *var. Boulardii and a recently developed prebiotic β-galactomannan, to prevent *E.coli *K88 infection using an in vitro model for pig intestinal cells, and consequently their ability to reduce pathogenic inflammation. These results may lead to in vivo trials to assess the suitability of probiotic *Scb *and prebiotic βGM as alternative to antibiotics growth promoters in animal production.

## List of abbreviations

APRIL: A proliferation-inducing ligand; BAFF: B-cell activating factor; βGM: β-galactomannan; CCL: chemokine: C-C motif) ligand; CXCL: chemokine: C-X-C motif) ligand; DCs: Dendritic cells; ETEC: Enterotoxigenic *Escherichia coli *K88; GM-CSF: Granulocyte/Macrophage Colony-Stimulating Factor; IECs: Intestinal epithelial cells; IL: Interleukin; NDOs: Non-digestible oligosaccharides; PAMPs: Pathogen associated molecular patterns; PRRs: Pattern recognition receptors; *Scb *(*Saccharomyces cerevisiae *var. Boulardii; TLR: Toll like receptor; TNFα: Tumor necrosis factor-α).

## Competing interests

JB is one of the inventors of the patent Salmosan^® ^WO2009/144070 A2 commercially licensed to Industrial Técnica Pecuaria (ITPSA, Barcelona, Spain). This does not alter the author's adherence to all the Veterinary Research policies on data collection and analysis, preparation of the manuscript or sharing data and materials. Other authors declare that they have no competing interests.

## Authors' contributions

RB conceived the study and carried out IECs and DCs host cell-pathogen co-cultures, molecular studies and immunoassays. GZ participated in IECs host cell-pathogen assays and molecular analysis. CC worked in DCs differentiation and co-culture. RL participated in experimental design and statistical analysis. PM, FM, JB and HS equally conceived, coordinated the study and helped to draft the manuscript. All authors read and approved the final manuscript.
